# Rehospitalizations Following Primary Percutaneous Coronary Intervention in Patients With ST‐Elevation Myocardial Infarction: Results From a Multi‐Center Randomized Trial

**DOI:** 10.1161/JAHA.117.005926

**Published:** 2017-08-05

**Authors:** Ernest Spitzer, Martina Frei, Serge Zaugg, Susanne Hadorn, Henning Kelbaek, Miodrag Ostojic, Andreas Baumbach, David Tüller, Marco Roffi, Thomas Engstrom, Giovanni Pedrazzini, Vladan Vukcevic, Michael Magro, Ran Kornowski, Thomas F. Lüscher, Clemens von Birgelen, Dik Heg, Stephan Windecker, Lorenz Räber

**Affiliations:** ^1^ Department of Cardiology Bern University Hospital Bern Switzerland; ^2^ Department of Interventional Cardiology Thoraxcenter Erasmus University Medical Center Rotterdam The Netherlands; ^3^ Clinical Trials Unit University of Bern Switzerland; ^4^ Institute of Social and Preventive Medicine University of Bern Switzerland; ^5^ Department of Cardiology Zealand University Hospital Roskilde Denmark; ^6^ Department of Cardiology Clinical Center of Serbia Belgrade Serbia; ^7^ William Harvey Research Institute Queen Mary University and Barts Heart Centre London United Kingdom; ^8^ Cardiology Department Triemlispital Zurich Switzerland; ^9^ Cardiocentro Lugano Switzerland; ^10^ Department of Cardiology Rigshospitalet Copenhagen Denmark; ^11^ Division of Cardiology University Hospital Geneva Switzerland; ^12^ Department of Cardiology TweeSteden Ziekenhuis Tilburg The Netherlands; ^13^ Rabin Medical Center Petach Tikva Israel; ^14^ Tel Aviv University Tel Aviv Israel; ^15^ Cardiology Department University Hospital Zurich Zurich Switzerland; ^16^ Thoraxcentrum Twente Twente University Enschede The Netherlands

**Keywords:** cardiac hospitalization, coronary artery disease, myocardial infarction, percutaneous coronary intervention, rehospitalization, Quality and Outcomes, Percutaneous Coronary Intervention, Revascularization, Coronary Artery Disease

## Abstract

**Background:**

Rehospitalizations (RHs) after ST‐elevation myocardial infarction carry a high economic burden and may deteriorate quality of life. Characterizing patients at higher risk may allow the design of preventive measures. We studied the frequency, reasons, and predictors for unplanned cardiac and noncardiac RHs in ST‐elevation myocardial infarction patients undergoing primary percutaneous coronary intervention.

**Methods and Results:**

In this post‐hoc analysis of the COMFORTABLE AMI (Comparison of Biolimus Eluted From an Erodible Stent Coating With Bare Metal Stents in Acute ST‐Elevation Myocardial Infarction; NCT00962416) trial including 1137 patients, unplanned cardiac and noncardiac RHs occurred in 133 (11.7%) and in 79 patients (6.9%), respectively, at 1 year. The most frequent reasons for unplanned cardiac RHs were recurrent chest pain without evidence of ischemia (20.4%), recurrent chest pain with ischemia and coronary intervention (16.9%), and ischemic events (16.9%). Unplanned noncardiac RHs occurred most frequently attributed to bleeding (24.5%), infections (14.3%), and cancer (9.1%). On multivariate analysis, left ventricular ejection fraction (22% increase in the rate of RHs per 10% decrease; *P*=0.03) and angiographic myocardial infarction Syntax score (34% increase per 10‐point increase; *P*=0.01) were independent predictors of unplanned cardiac RHs. Age emerged as the only independent predictor of unplanned noncardiac RHs. Regional differences for unplanned cardiac RHs were observed.

**Conclusions:**

Among ST‐elevation myocardial infarction patients undergoing primary percutaneous coronary intervention in the setting of a randomized, clinical trial, unplanned cardiac RHs occurred in 12% with recurrent chest pain being the foremost reason. Unplanned noncardiac RHs occurred in 7% with bleeding as the leading cause. Left ventricular ejection fraction and Syntax score were independent predictors of unplanned cardiac RHs and identified patient subgroups in need for improved secondary prevention.

**Clinical Trial Registration:**

URL: http://www.clinicaltrials.gov. Unique identifier: NCT00962416.

**Clinical Perspective**

**What is New?**

- Among patients undergoing percutaneous coronary intervention for ST‐elevation myocardial infarction, unplanned cardiac rehospitalizations at 1 year occurred in 12%, representing approximately two thirds of all unplanned rehospitalizations.
- The most common reason for unplanned cardiac rehospitalizations was recurrent chest pain, which was categorized by treating physicians as noncoronary in one third of cases.
- Syntax myocardial infarction score and left ventricular ejection fraction were independent predictors of unplanned cardiac rehospitalizations at 1 year.
- Unplanned noncardiac rehospitalizations at 1 year occurred in 7% of patients with bleeding as the leading cause and age emerging as the only independent predictor identified.

**What are the Clinical Implications?**

- Myocardial infarction Syntax score and predischarge ejection fraction assessment may help in identifying patients at increased risk for unplanned cardiac rehospitalizations and who could benefit from closer monitoring after percutaneous coronary intervention for ST‐elevation myocardial infarction.

## Introduction

Rehospitalizations (RHs) after myocardial infarction (MI) are the aftermath of in‐hospital quality of care, postdischarge management, and an individual's vulnerability.^1^, ^2^ Up to one fifth of patients who survive to hospital discharge will be readmitted within 1 month,^3^, ^4^ with the consequent economic burden as well as impact on quality of life and long‐term prognosis. Identifying the characteristics associated with RHs could aid in stratifying patients according to their risk of RHs and assist physicians in optimizing management following discharge.^5^

Although extensive data have been published regarding RHs after acute MI,^5^ RHs after primary percutaneous coronary intervention (PCI) in patients presenting with ST‐segment elevation MI (STEMI) have not been systematically studied, and available data are limited to 30 days of follow‐up.^2^, ^4^ Moreover, predictors of RHs have not been consistent throughout studies, which is partially explained by the diverse types of RHs reported (eg, all cause, cardiac related), as well as the quality and granularity of data expected based on the study design.^5^ In addition, regional differences in the frequency emerged in multinational studies, underscoring the impact of health systems and local practices on RHs.^2^

Planned RHs post‐PCI for STEMI may reflect good clinical practice, given that they relate to clinical decisions to better diagnose or treat patients. Such decisions could occur before (eg, hip replacement), during (eg, staged PCI), or after (eg, implantation of a cardioverter defibrillator) the index hospitalization. Consequently, examining unplanned RHs may better characterize patients at increased risk. Our aim was to investigate the frequency, reasons, and predictors of unplanned cardiac and noncardiac RHs following primary PCI in STEMI patients.

## Methods

### Study Design and Patient Population

We performed a post‐hoc analysis of data from the COMFORTABLE AMI trial (Comparison of Biolimus Eluted From an Erodible Stent Coating With Bare Metal Stents in Acute ST‐Elevation Myocardial Infarction; NCT00962416). The study design has been published elsewhere.^6^ In brief, this was a multicenter, randomized, assessor‐blinded, superiority trial, in which 1161 STEMI patients undergoing primary PCI at 11 sites in 5 countries in Europe and Israel between September 19, 2009 and January 25, 2011 were assigned to a biolimus drug‐eluting stent (BioMatrix; Biosensors Europe SA, Morges, Switzerland) or to a bare metal stent (Gazelle; Biosensors Europe SA, Morges, Switzerland).

Patients were eligible for randomization if they were aged 18 years or older with symptom onset within 24 hours, electrocardiographic criteria for STEMI, and angiographic evidence of at least 1 culprit lesion within the infarct vessel. There were no limits regarding the number of treated lesions, vessels, or complexity. Exclusion criteria were the presence of mechanical complications of acute MI, known allergy to any study medication, use of vitamin K antagonists, planned surgery unless dual antiplatelet therapy could be maintained during the perisurgical period, history of bleeding diathesis or known coagulopathy, pregnancy, participation in another trial before reaching the primary end point, inability to provide informed consent, and noncardiac comorbid conditions with life expectancy of less than 1 year.

For the purpose of the current analysis, all randomized, controlled trial participants were included, except for patients not surviving index hospitalization, or presenting with out‐of‐hospital death as first event at follow‐up. The study complied with the Declaration of Helsinki and was approved by the institutional ethics committee at each participating hospital. All patients included provided written informed consent.

### Procedures

After randomization, all patients were treated according to current guidelines and the following co‐interventions were indicated: (1) Thrombus aspiration was recommended in all patients whenever aspiration was deemed technically feasible; (2) complete revascularization of all lesions within the infarct vessel had to be performed with the randomly allocated study stent; and (3) nonculprit vessels were treated by default by biolimus‐eluting stent at baseline and during follow‐up.

Acetylsalicylic acid (≥250 mg) was administered before the procedure. In the absence of contraindications, patients received an initial dose of prasugrel 60 mg (including patients preloaded with clopidogrel) followed by a daily dose of 10 mg. When prasugrel was contraindicated, clopidogrel was administered at a loading dose of 600 mg, followed by a dose of 75 mg twice‐daily for 7 days, followed by a maintenance dose of 75 mg once‐daily. Dual antiplatelet therapy was prescribed for the duration of at least 1 year in all patients.

### Data Collection

All RHs were prospectively collected by dedicated research personnel in the frame of the trial's severe adverse event reporting process throughout 2 years. Paper forms were centrally collected and entered into a dedicated database (Cardiobase, Clinical Trials Unit, and Department of Cardiology, Bern University Hospital, Bern, Switzerland).

### Study End Point Definitions

RHs were defined as any visit to the emergency department for more than 6 hours or any hospital admission. Unplanned RHs were defined as unscheduled visits. In all instances, the main reasons for RH were ascertained in predefined categories (see Table S1 and Table 1). Two investigators (E.S., M.F.) reviewed independently all severe adverse events and adjudicated RHs. Disagreements were adjudicated by a third investigator (L.R.). When several factors contributed to RHs, all were adjudicated, and a predefined study‐specific hierarchical rule was used to assign the main reason of RH (see Tables S2 and S3). MI Syntax score was determined as previously described in detail.^7^ Recurrent ischemic events were defined according to the previously published study protocol.^6^ Recurrent chest pain and ischemia with or without intervention was defined as chest pain attributed to angiographically significant stenosis (with or without intervention) or positive ischemia testing during the unscheduled hospitalization. Chest pain in the absence of angiographic significant stenosis or negative ischemia testing was defined as chest pain without evidence of ischemia.

**Table 1:** Reasons for Unplanned Rehospitalizations After Primary PCI for STEMI (Including Multiple Events)

	No. of RHs	% of RHs
Unplanned cardiac rehospitalizations		
Recurrent chest pain without evidence of ischemia^a^	35	20.35
Recurrent chest pain with ischemia and subsequent intervention^b^^,^ c	29	16.86
Ischemic events (myocardial infarction and/or stent thrombosis)	29	16.86
Heart failure	28	16.28
Recurrent chest pain with ischemia with no coronary intervention^b^	23	13.37
Tachyarrhythmias	11	6.40
Miscellany	9	5.23
Bradyarrhythmias	5	2.91
Pericarditis	3	1.74
Total	172	100
Unplanned noncardiac rehospitalizations		
All bleeding	24	24.49
Miscellany^d^	16	16.33
Infections (including sepsis)	14	14.29
Neoplasms^e^	9	9.18
Abdominal pathology^e^	8	8.16
Cerebrovascular event	8	8.16
Major vascular complication (excluding bleeding)	4	4.08
Respiratory pathology^e^	3	3.06
Urogenital pathology^e^	3	3.06
Surgery: orthopedics	2	2.04
Surgery: abdominal	2	2.04
Surgery: miscellany	2	2.04
Acute renal failure (including \decompensation of CKD)	2	2.04
Surgery: oncology (including biopsies)	1	1.02
Total	98	100

CKD indicates chronic kidney disease; PCI, percutaneous coronary intervention; RH, rehospitalization; STEMI, ST‐elevation myocardial infarction.

a Among 35 RHs, 8 included repeat coronary angiographies, 6 noninvasive stress tests, and 2 noninvasive myocardial perfusion tests.

b Stable or unstable angina.

c Percutaneous coronary intervention or coronary bypass graft surgery.

d Accident, pupura, pemphigus, drug toxicity, allergies, gout, psychiatric disorder, or vertigo.

e Diagnosis or nonsurgical treatment.

### Statistical Analysis

Continuous variables are expressed as means±SD or medians with 25th and 75th percentiles. *P* values were derived using *t* tests in the former and Mann–Whitney *U* tests in the latter. Categorical data are expressed as frequencies and percentages and are compared using the χ^2^ and Fischer's exact tests. For nested variables in lesion‐level data, *P* values from general and generalized mixed models were used. Poisson regressions, or negative binomial regressions in case of overdispersion, were used to calculate rates of RH. To identify risk factors, first, missing values of these factors were multiple‐imputed using chained equations (40 data sets created): body mass index, left ventricular ejection fraction, SYNTAX score, hemoglobin, low‐density lipoprotein (LDL), renal insufficiency, hypertension, and history of malignancy; note that also nonmissing information (age, sex, and cardiac unplanned and noncardiac unplanned RH) were used in these equations. Second, Poisson regressions or negative binomial regressions were performed on each of these 40 data sets and estimates were combined using Rubin's rule. Third, predictors included were all variables with a univariable effect of *P*<0.05 on either patients with any cause of unplanned cardiac RHs or patients with any cause of noncardiac RHs. One single cause was determined for each RH, and predictors analyses were done considering multiple RHs. Baseline and procedural characteristics were compared at patient level (ie, patients without versus with 1 or more RHs). Kaplan–Meier curves for time to first (unplanned cardiac or unplanned noncardiac) RH were constructed. All *P* values shown are from 2‐sided tests, and the level of statistical significance was set at 0.05. Analyses were performed using Stata software (version 13; StataCorp LP, College Station, TX).

## Results

Among the 1161 STEMI patients enrolled in the trial, a total of 1137 (97.9%) were eligible for this subanalysis. The patient flow chart is provided in Figure S1. Unplanned cardiac RHs amounted to 172 episodes in 133 (11.7%) patients at 1 year, and 98 episodes of unplanned noncardiac RHs were observed in 79 (6.9%) patients at 1 year (Figure). Reasons for unplanned cardiac and noncardiac RHs are presented in Table 1. A total of 64 planned rehospitalizations were identified and details are provided in Table S4.

**Figure 1:**
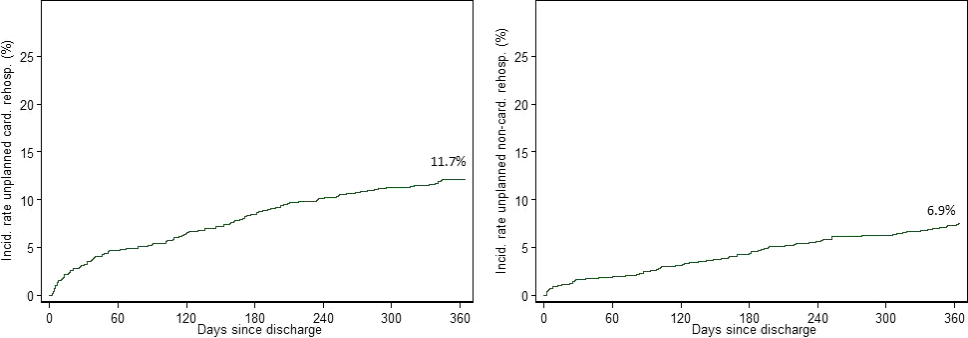
Kaplan–Meier curves showing the incidence rate of unplanned cardiac and noncardiac rehospitalizations within 1 year.

### Baseline and Procedural Characteristics

Baseline and procedural characteristics are summarized in Table 2. Patients with at least 1 episode of unplanned cardiac RH were older (62.5 versus 59.7 years; *P*=0.012) and had similar comorbidities with the exception of higher prevalence of renal failure (11.5% versus 6.3%; *P*=0.042) as compared with patients without unplanned cardiac RH. Baseline LDL cholesterol (3.1 versus 3.3 mmol/L; *P*=0.037) and left ventricular ejection fraction were lower (45% versus 50%; *P*=0.006), whereas Syntax MI score was higher (16 versus 13 points; *P*=0.002) in the unplanned cardiac RH cohort.

**Table 2:** Baseline and Procedural Characteristics (Including First Events Only)

	Overall	No RHs	Unplanned Cardiac RHs	Unplanned Noncardiac RHs	*P* Value	*P* Value
	N=1137	N=925	N=133	N=79	Cardiac vs No RHs	Noncardiac vs No RHs
Age, y	60.5 (52.1; 69.0)	59.7 (51.4; 67.8)	62.5 (53.3; 71.2)	67.3 (59.5; 77.5)	0.012	0.001
Female sex (%)	228 (20.1)	173 (18.7)	25 (18.8)	30 (38.0)	1.00	0.001
Body mass index, kg/m^2^	26.7 (24.3; 29.4)	26.8 (24.5; 29.5)	26.8 (24.0; 29.7)	25.7 (23.4; 28.7)	0.50	0.013
Heart rate, bpm	75.0 (65.0; 85.0)	75.0 (65.0; 85.0)	76.0 (64.0; 87.0)	75.0 (63.3; 84.3)	0.90	0.51
Family history of coronary artery disease (%)	366 (32.8)	312 (34.1)	35 (27.1)	19 (26.0)	0.13	0.20
Diabetes mellitus (%)	168 (14.8)	138 (14.9)	21 (15.8)	9 (11.4)	0.80	0.51
Current smoker (%)	568 (50.5)	466 (51.0)	64 (48.1)	38 (48.7)	0.58	0.73
Hypertension (%)	529 (46.5)	421 (45.5)	61 (45.9)	47 (59.5)	1.00	0.02
Hypercholesterolemia^a^	645 (57.0)	529 (57.5)	71 (53.8)	45 (57.0)	0.45	1.00
Previous CAD^b^	79 (6.9)	66 (7.1)	9 (6.8)	4 (5.1)	1.00	0.65
Previous myocardial infarction (%)	58 (5.1)	49 (5.3)	6 (4.5)	3 (3.8)	0.84	0.79
Previous PCl (%)	45 (4.0)	37 (4.0)	5 (3.8)	3 (3.8)	1.00	1.00
Previous CABG (%)	14 (1.2)	10 (1.1)	3 (2.3)	1 (1.3)	0.22	0.60
History of congestive heart failure (%)	8 (0.7)	8 (0.9)	0 (0.0)	0 (0.0)	0.61	1.00
History of stroke or TIA (%)	35 (3.1)	25 (2.7)	3 (2.3)	7 (8.9)	1.00	0.010
History of malignancy (%)	53 (4.7)	41 (4.4)	5 (3.8)	7 (8.9)	1.00	0.09
Chronic obstructive lung disease (%)	31 (2.7)	23 (2.5)	4 (3.0)	4 (5.1)	0.77	0.16
History of gastrointestinal bleeding (%)	16 (1.4)	13 (1.4)	2 (1.5)	1 (1.3)	1.00	1.00
Clinical relevant valvular disease (%)	3 (0.3)	2 (0.2)	0 (0.0)	1 (1.3)	1.00	0.22
Renal failure^c^	83 (7.5)	57 (6.3)	15 (11.5)	11 (13.9)	0.04	0.02
Time from symptom onset to balloon inflation, min	232.0 (162.0; 380.0)	228.0 (162.0; 372.0)	250.0 (161.0; 436.0)	246.0 (164.0; 416.0)	0.32	0.87
Resuscitation preceding hospital arrival (%)	23 (2.0)	20 (2.2)	1 (0.8)	2 (2.5)	0.50	0.69
Killip III or IV (III or IV) (%)	14 (1.2)	10 (1.1)	3 (2.3)	1 (1.3)	0.22	0.60
Left ventricular function, %	50.0 (40.0; 60.0)	50.0 (42.0; 60.0)	45.0 (40.0; 55.0)	50.0 (44.3; 59.8)	0.006	0.64
Treatment of LAD (%)	469 (41.3)	377 (40.8)	63 (47.4)	29 (36.7)	0.16	0.55
Bifurcation treatment (%)	99 (8.7)	81 (8.8)	14 (10.5)	4 (5.1)	0.52	0.40
Multivessel treatment (%)	65 (5.7)	49 (5.3)	12 (9.0)	4 (5.1)	0.11	1.00
SYNTAX MI score, points	13.0 (9.0; 20.5)	13.0 (9.0; 20.0)	16.0 (10.5; 22.0)	12.0 (9.0; 20.0)	0.002	0.49
Length of stay at index hospitalization, d	3.0 (2.0; 5.0)	3.0 (2.0; 5.0)	3.0 (2.0; 4.0)	3.0 (1.0; 4.0)	0.81	0.33
Postprocedural TIMI flow (%)					0.82	0.78
TIMI flow 0 to 2	51 (4.5)	42 (4.6)	5 (3.8)	4 (5.1)	0.82	0.78
TIMI flow 3	1084 (95.5)	881 (95.4)	128 (96.2)	75 (94.9)	0.82	0.78
Hemoglobin baseline, g/L	141.0 (132.0; 151.0)	142.0 (133.0; 152.0)	140.0 (130.0; 150.0)	137.0 (124.1; 147.3)	0.05	0.001
Thrombocytes baseline, g/L	234.0 (196.0; 273.0)	233.0 (196.0; 269.0)	233.5 (190.5; 290.5)	243.5 (190.5; 292.5)	0.49	0.17
LDL baseline, mmol/L	3.3 (2.6; 4.0)	3.3 (2.7; 4.0)	3.1 (2.5; 3.9)	2.8 (2.3; 3.7)	0.04	0.002
Country (%)						
Serbia	167 (14.7)	158 (17.1)	4 (3.0)	5 (6.3)		
Switzerland	547 (48.1)	447 (48.3)	58 (43.6)	42 (53.2)		
United Kingdom	107 (9.4)	89 (9.6)	12 (9.0)	6 (7.6)		
Denmark	170 (15.0)	119 (12.9)	35 (26.3)	16 (20.3)		
Israel	47 (4.1)	30 (3.2)	14 (10.5)	3 (3.8)		
The Netherlands	99 (8.7)	82 (8.9)	10 (7.5)	7 (8.9)		

Results presented as median (25%, 75% percentile) or number (%). *P* values from the Kruskal–Wallis test or Fisher's exact test. CABG indicates coronary artery bypass graft surgery; CAD, coronary artery disease; LAD, left anterior descending artery; PCI, percutaneous coronary intervention; RH, rehospitalization; TIA, transient ischemic attack; TIMI, Thrombolysis In Myocardial Infarction.

a Total cholesterol >5.0 mmol or 190 mg/dL or under treatment.

b Includes previous MI, PCI, or CABG.

c Glomerular filtration rate lower than 60 mL/min.

Patients readmitted for unplanned noncardiac reasons were older (67.3 versus 59.7 years; *P*=0.001), more frequently women (38% versus 18.7%; *P*=0.001), and had a lower body mass index (25.7 versus 26.8 kg/m^2^; *P*=0.013) as compared with patients without unplanned RHs. Comorbidities were more frequent in patients with unplanned noncardiac RHs, including higher prevalence of hypertension (59.5% versus 45.5%; *P*=0.019), history of stroke or transient ischemic attack TIA (8.9% versus 2.7%; *P*=0.01), and renal failure (13.9% versus 6.3%; *P*=0.018). Moreover, unplanned noncardiac RH patients had a lower baseline hemoglobin (13.7 versus 14.2 g/dL; *P*=0.001) and LDL cholesterol (2.8 versus 3.3 mmol/L; *P*=0.002).

### Reasons of Unplanned Rehospitalizations According to Stent Assignment

We observed no significant differences in the overall rate of rehospitalization among patients assigned to bare‐metal stents when compared with those assigned to a biolimus‐eluting stent, except for recurrent chest pain with ischemia and subsequent intervention, which was more frequently the reason for rehospitalization in patients treated by a bare‐metal stent (23.4% versus 9.0% [percentage of all RHs]; *P*=0.01). Moreover, when analyzing this reason of rehospitalization at a patient level, 4.4% of the patients assigned to the bare‐metal stent group exhibited 1 or more RHs versus 1.1% in the biolimus‐eluting stent group (incidence rate ratio, 3.1; 95% CI, 1.28–7.45). Detailed reasons for RH among stent groups are provided in Table S5.

### Predictors of Unplanned RHs

For the purpose of the multivariate analysis, we estimated the rate of unplanned RHs within 1 year, accounting for multiple events. The rate of unplanned cardiac RHs was 0.15 per patient per year (95% CI, 0.13–0.18), whereas the rate of unplanned noncardiac RHs was 0.09 per patient per year (95% CI, 0.07–0.11). Predictors of 1‐year unplanned cardiac RHs after adjustment for baseline and procedural characteristics are presented in Tables 3 and 4. Each 10% decrease in left ventricular ejection fraction was associated with a 22% increase in the rate of unplanned cardiac RHs (95% CI, 1.02–1.46, *P*=0.03), and each 10‐unit increase in Syntax‐MI score preceding primary PCI was associated with a 34% increase (95% CI, 1.07–1.68; *P*=0.01). No other independent predictors of unplanned cardiac RHs were identified.

**Table 3:** Predictors of Unplanned Cardiac Rehospitalizations Within 1 Year After PCI for STEMI (Including Multiple Events)

	Rate		95% CI
Overall Rate: No. of Rehospitalizations Per Patient Per Y	0.15		0.13 to 0.18
Predictors	Rate Ratio	*P* Value	95% CI
Age (per 10 y)	1.02	0.77	0.87 to 1.21
Left ventricular ejection fraction (%) (per 10‐unit decrease)	1.22	0.03	1.02 to 1.46
MI Syntax score (per 10‐unit increase)	1.34	0.01	1.07 to 1.68
Hemoglobin baseline, g/L	1.00	0.53	0.98 to 1.01
LDL baseline, mmol/L	0.88	0.19	0.74 to 1.06
Renal failure (GFR <60 mL/min)	1.18	0.61	0.63 to 2.21

Results from multivariable negative binomial regression with estimated rates of rehospitalisation after multiple imputation of missing data using chained equations. Estimates combined from 40 imputed data sets using Rubin's rule. GFR indicates glomerular infiltration rate; LDL, low‐density lipoprotein; MI, myocardial infarction; PCI, percutaneous coronary intervention; ST‐elevation myocardial infarction.

**Table 4:** Predictors of Unplanned Noncardiac Rehospitalizations Within 1 Year After PCI for STEMI (Including Multiple Events)

	Rate		95% CI
Overall Rate: No. of Rehospitalizations Per Patient Per Y	0.09		0.07 to 0.11
Predictors	Rate Ratio	*P* Value	95% CI
Age (per 10 y)	1.43	0.001	1.15 to 1.77
BMI (per 10 units)	0.86	0.59	0.51 to 1.47
Hemoglobin baseline, g/L	1.00	0.56	0.98 to 1.01
LDL baseline, mmol/L	0.83	0.11	0.65 to 1.05
Male sex	0.63	0.08	0.38 to 1.05
Hypertension	1.41	0.15	0.88 to 2.28
Renal failure (GFR <60 mL/min)	0.98	0.96	0.50 to 1.95
History of malignancy	1.58	0.23	0.75 to 3.34

Results from multivariable negative binomial regression with estimated rates of rehospitalisation after multiple imputation of missing data using chained equations. Estimates combined from 40 imputed data sets using Rubin's rule. BMI indicates body mass index; GFR, glomerular infiltration rate; LDL, low‐density lipoprotein; PCI, percutaneous coronary intervention; ST‐elevation myocardial infarction.

Among patients who presented for unplanned noncardiac RHs, age was the only independent predictor. A 10‐year increase of age was associated with a 43% increase in the rate of unplanned noncardiac RHs (95% CI, 1.15–1.77; *P*=0.001).

### Rates of Unplanned RHs at Country Level

Rates of unplanned cardiac and noncardiac RHs stratified by countries are presented in Table 5. Among the 6 participating countries, the rate of unplanned cardiac RHs per patient per year was highest in Israel (0.40; 95% CI, 0.22–0.74) and Denmark (0.26; 95% CI, 0.18–0.38) and lowest in Serbia (0.02; 95% CI, 0.01–0.07). Rates of RHs among The Netherlands, Switzerland, and the United Kingdom were comparable. Regional rates of readmission were more homogenous for unplanned noncardiac RHs, ranging from 0.07 (95% CI, 0.04–0.16) in the United Kingdom to 0.12 (95% CI 0.07–0.19) in Denmark. Finally, Serbia presented the lowest rate of readmissions for unplanned noncardiac causes (0.03; 95% CI, 0.01–0.07).

**Table 5:** Country‐Specific Rates of Unplanned Rehospitalizations (Including Multiple Events)

	Rate	95% CI
Unplanned cardiac rehospitalizations (number per patient per y)		
Overall	0.15	0.13 to 0.18
Serbia	0.02	0.01 to 0.07
Switzerland	0.14	0.11 to 0.18
United Kingdom	0.13	0.07 to 0.24
Denmark	0.26	0.18 to 0.38
Israel	0.40	0.22 to 0.74
The Netherlands	0.13	0.07 to 0.24
Unplanned noncardiac rehospitalizations (number per patient per y)		
Overall	0.09	0.07 to 0.11
Serbia	0.03	0.01 to 0.07
Switzerland	0.09	0.07 to 0.12
United Kingdom	0.07	0.04 to 0.16
Denmark	0.12	0.07 to 0.19
Israel	0.11	0.04 to 0.28
The Netherlands	0.09	0.04 to 0.19

## Discussion

The main findings of this analysis on the frequency, reasons, and predictors of unplanned cardiac and noncardiac RHs post‐PCI in patients presenting with STEMI can be summarized as follows:

1. Unplanned cardiac RHs at 1 year occurred in 12% of patients representing approximately two thirds of all unplanned RHs. When accounting for multiple events, the annual RH rate per patient was 0.15.
2. The most common reason for unplanned cardiac RHs post‐PCI for STEMI was recurrent chest pain, which was categorized by treating physicians as noncoronary origin in one third of cases.
3. Syntax MI score and left ventricular ejection fraction were independent predictors of unplanned cardiac RHs at 1 year and may aid in the identification of patients at risk for unplanned cardiac RHs.
4. Unplanned noncardiac RHs at 1 year occurred in 7% of patients with bleeding as the leading cause and age emerging as the only independent predictor identified.

The largest evidence available on RHs after acute MI is derived from national data sets from the United States including more than 500 000 patients, in which one fifth were rehospitalized within 30 days.^8^ Our lower rates of RH are explained by the selective inclusion of unplanned RHs only, as opposed to all‐cause RHs,^8^ the lower risk of RHs expected in a randomized, controlled trial with exclusion of very‐high‐risk patients (eg, baseline critical condition, high bleedings risk)^9^ and the previously reported lower rates of readmission in European countries, when compared with the United States.^2^ In the Assessment of Pexelizumab in Acute Myocardial Infarction trial, which enrolled over 5000 STEMI patients in 17 countries, the 30‐day all‐cause readmission was 11.3%.^2^ In several countries, the odds of RHs where less than half when compared with the United States, including Italy, Germany, Canada, Portugal, and The Netherlands. In line with our findings, Switzerland was associated with a numerically lower rate of RHs and Denmark with a numerically higher rate. Further confirmation of regional variability stems from a recent study on 30‐day all‐cause RHs post‐PCI in Italy, with showed a rate as low as 4.7%.^10^ These differences may relate to patient characteristics and local practices (ie, access site, referral habits). Given that potential associated factors were not systematically assessed, we may infer about other determinants, such as: (1) accessibility to emergency department, and whether patients visited first a general practitioner or not; (2) physicians counseling regarding chest pain recurrence and conditions that should prompt consultation; and (3) differences in the threshold for readmission among countries. Moreover, the possibility that true differences in the rate of complications following primary percutaneous coronary intervention for STEMI or that a misrepresentation of event rates exist, may stimulate further research in this field. Given that comparable clinical outcomes (ie, major adverse cardiac events) among countries were observed, true differences in complications other than major adverse cardiac events following STEMI represents an unlikely explanation.

### Reasons for Unplanned Cardiac and Noncardiac RH

Our results indicate that recurrent chest pain is the most common reason for unplanned RHs after PCI for STEMI, amounting to two thirds of unplanned cardiac readmissions within 1 year. Even though 70% of those RHs justified a revascularization procedure or specialized care, the remainders comprised a subgroup of patients in which an opportunity to reduce rates of RHs exist. Chest pain without evidence of ischemia (ie, noncoronary) comprises a wide range of diagnoses with varying risk and need for RH, from life‐threatening conditions (eg, aortic dissection, pulmonary embolism, and acute coronary syndrome) to low‐risk ailments (eg, musculoskeletal pain, gastroesophageal reflux, and hypertensive cardiomyopathy). Proper algorithms in emergency departments coupled with noninvasive imaging techniques and laboratory tests with high negative predictive value, could potentially avoid unnecessary RHs, with the consequent reduction in health costs. Conversely, the universal availability of such tests might be restrained because of economic constraints. In our study, 60% of patients were discharged based on normal biomarkers and absence of electrocardiographic changes, whereas 23% underwent coronary catheterization. The relevance of recurrent chest pain without the need for reintervention has been highlighted in PCI registries,^11^, ^12^ 1 of which reported repeat revascularizations in less than 1 in 8 patients admitted with chest pain, and suggested that low‐risk chest pain may be effectively evaluated in the outpatient or observational settings. Patient counseling as to how to adequately react to a pain episode may also translate in less cardiac RHs attributed to chest discomfort.

We observed that bleeding was the most frequent noncardiac cause of RHs and not unexpectedly in this context, age emerged as an independent predictor in our analysis. Other investigators have reported the frequency of bleeding post‐PCI for STEMI^13^, ^14^; however, this study is the first to show bleeding as a leading cause. Acute coronary syndrome patients are at a high risk for bleeding events partly related to the administration of more‐potent antiplatelet therapy.^15^, ^16^ Bleeding post‐PCI is relevant considering its association with an increased risk of death at long‐term follow‐up.^17^ Among patients undergoing PCI, the most frequent bleeding site is the gastrointestinal tract.^18^ Noteworthy, increasing age, previous gastrointestinal bleed, history of malignancy, smoking, and triple antithrombotic therapy were reportedly independent predictors of gastrointestinal bleeding.^17^ Upper gastrointestinal bleeding appears preventable, in part, by administration of proton pump inhibitors. Indeed, a recent study confirmed a significant reduction in gastrointestinal bleeding events by routine administration of proton pump blockers in PCI patients receiving dual antiplatelet therapy.^19^ Furthermore, the evaluation of bleeding risk by using dedicated scores may aid in identifying patients in whom potent P2Y12 administration or a prolonged dual antiplatelet therapy duration should be omitted.^20^, ^21^

### Predictors of Unplanned Cardiac and Noncardiac RHs

Early RHs carry a significant economic burden,^12^ and reducing the number of RHs by improving quality of care specifically in patients at high risk could lend to a better utilization of resources. Notwithstanding, such an undertaking may turn out complex. For instance, early physician follow‐up have not been associated with lower 30‐day RH rates in patients discharged after non‐STEMI.^22^

Baseline and procedural characteristics associated with unplanned RHs vary based on whether the cause of RH is cardiac or noncardiac. Interestingly, comorbidities, such as hypertension, previous stroke or transient ischemic attack, history of malignancy, and anemia, were associated only with unplanned noncardiac RHs, whereas lower left ventricular systolic function and higher MI Syntax score were associated only with unplanned cardiac RHs. A common characteristic of patients being readmitted for both cardiac and noncardiac reason was a significantly lower LDL level measured before initialization of statin therapy. Although not emerging as an independent predictor, the association of low LDL and unplanned RHs adds to previous observations including an association between low LDL levels and increased total and cardiovascular mortality in elderly^23^, ^24^ and acutely diseased individuals,^25^ as well as an increased risk of cancer.^26^ Based on the reportedly consistent positive effect of LDL lowering by statins in patients with coronary artery disease, it can be assumed that a reverse causal relationship is responsible for our observation.

Few independent predictors could be identified following multivariate analysis. Age was the only one predicting readmission for noncardiac reasons and left ventricular function and Syntax MI score before revascularization. The impact of ejection fraction on the prognosis of survivors of acute MI is well established.^27^, ^28^ Conversely, this is the first report on an association between the MI Syntax score with unplanned cardiac RHs. A previous large‐scale study found an association on multivessel disease and readmissions, a finding that was no longer present after the exclusion of planned revascularization procedures, which were not considered in our investigation. The Syntax score quantifies angiographic disease complexity and was shown to predict patient‐ and device‐oriented major adverse cardiac events during follow‐up in various clinical setting, including STEMI.^7^, ^29^ Although full revascularization was recommended within the initial hospital stay, significant lesions may persisted and were treated conservatively, subsequently leading to unplanned cardiac RH, an assumption that is supported by a high contribution of repeat revascularization procedures in previously untreated vessel segments. In the absence of information of the completeness of revascularization (ie, by assessing the residual Syntax score), we can only speculate as to whether a more complete revascularization at the index procedure or early after primary PCI could attenuate unplanned cardiac RHs, and thereby safe costs, as it was observed in previous studies.^30^ The total number of rehospitalization events in this cohort throughout 1 year did not allow to investigate predictors for early (<30 days) versus late (>30 days) rehospitalizations (cardiac and noncardiac).

In this study, patients receiving biodegradable polymer biolimus‐eluting stents were compared with patients receiving bare‐metal stents. The primary composite endpoint of cardiac death, target vessel MI and target lesion revascularization, occurred more frequently in patients treated with bare‐metal stents.^31^, ^32^ Overall, we observed no significant difference in the rate of unplanned cardiac or noncardiac rehospitalizations among groups. Nevertheless, and in line with the main report of the trial, recurrent chest pain with ischemia and subsequent intervention was more frequently observed in the bare‐metal stent group (4.4% versus 1.1%).

### Limitations

First, although COMFORTABLE AMI was designed as an all‐comers STEMI trial, ≈40% of patients presenting for STEMI were not included in the trial, leading to an underestimation of RH frequency, and the reasons and associated predictors of unplanned RH potentially differ. This is supported by a report that showed a higher rate of major adverse cardiovascular events in patients excluded from the COMFORTABLE AMI trial.^9^ Second, we did not record attendance to rehabilitation after primary PCI, a variable that is hypothetically attenuating the key end point of this study. Third, when multiple diagnoses coexisted in a hospitalization, we defined hierarchies that would allow categorization based on the cause of admission. These hierarchies may not be applicable to other settings. Fourth, because of the limited number of events and study subjects, we did not include country of enrollment in the predictors model. Finally, reasons for RH were ascertained by review of severe adverse events listings and narratives; consequently, adjudication was limited to the information available.

## Conclusion

Among STEMI patients undergoing primary PCI in the setting of a randomized, clinical trial, unplanned cardiac RHs occurred in 12%, with recurrent chest pain being the foremost reason. Unplanned noncardiac RHs occurred in 7% with bleeding as the leading cause. Left ventricular systolic function and Syntax score were independent predictors of unplanned cardiac RHs and identified patient subgroups in need for improved secondary prevention.

## Sources of Funding

Dr Windecker was supported by the Swiss National Science Foundation (33CM30‐124112 and 310030‐118353). Dr Spitzer received a Research Fellowship of the European Association of Percutaneous Cardiovascular Interventions (EAPCI) of the European Society of Cardiology and a Research Grant of the Spanish Society of Cardiology. The COMFORTABLE trial was supported by an unrestricted research grant from Biosensors Europe SA, Switzerland.

## Disclosures

Clinical Trials Unit Bern, which is part of the University of Bern, has a staff policy of not accepting honoraria or consultancy fees. Dr Windecker has received research contracts to the institution from Abbott, Boston Scientific, Biosensors, Biotronik, Cordis, and Medtronic. The other authors report no conflicts. Dr Räber has received grants to the institution from St. Jude Medical. Dr von Birgelen has received speakers honoraria from AstraZeneca, and his institution has received research grants from AstraZeneca, Biotronik, Boston Scientific, and Medtronic. The other authors report no conflict with regards to the content of this manuscript.

## Supporting information


**Table S1.** List of Reasons for Rehospitalizations
**Table S2.** Hierarchy Applied When Multiple Reasons for Unplanned Rehospitalizations Coexisted
**Table S3.** Hierarchy Applied When Multiple Reasons for Planned Rehospitalizations Coexisted
**Table S4.** Reasons for Planned Rehospitalizations After PCI for STEMI Within 1 Year
**Table S5.** Reasons for Unplanned Rehospitalizations After Primary PCI for STEMI Separate for Patients Randomized to Bare‐Metal Stent BMS and to Biolimus Drug‐Eluting Stent BES (Including Multiple Events)
**Figure S1.** Patient flow chart.
