# Supplementary material for: Rehospitalizations Following Primary Percutaneous Coronary Intervention in Patients With ST‐Elevation Myocardial Infarction: Results From a Multi‐Center Randomized Trial
Source: J Am Heart Assoc. 2017 Aug 5;6(8):e005926. doi: 10.1161/JAHA.117.005926 (PMC5586438; doi:10.1161/JAHA.117.005926)

Supplemental Material

Table S1. List of reasons for rehospitalizations

**Cardiac rehospitalizations**
Staged procedure or planned repeat angiography
Recurrent chest pain without evidence of ischemia*
Recurrent chest pain with ischemia but no intervention†
Recurrent chest pain with ischemia and coronary intervention†,‡
Ischemic events (myocardial infarction and/or stent thrombosis)
Bradyarrhythmias
Tachyarrhythmias
Planned implantation of a cardioverter defibrillator
Heart failure
Heart surgery due to valvular disease
Pericarditis
Other
**Non-cardiac rehospitalizations**
Cerebro-vascular event
All bleeding
Major vascular complication (excluding bleeding)
Acute renal failure (including decompensation of chronic kidney disease)
Respiratory pathology§
Abdominal pathology§
Urogenital pathology§
Neoplasms§
Infections (including sepsis)
Surgery: vascular (aorta, peripheral arteries)
Surgery: orthopedics
Surgery: abdominal
Surgery: urogenital
Surgery: oncology (including biopsies)
Surgery: other than the above
Non-cardiac vascular death
Non cardiovascular death
Other‖

*Chest pain without confirmed source or manifestation of ischemia after diagnostic tests
†Chest pain from cardiac origin with confirm source or manifestation of ischemia (stable or unstable angina)
‡Percutaneous coronary intervention or coronary artery bypass graft surgery
§Diagnosis or non-surgical treatment
‖Accident, pupura, pemphigus, drug toxicity, allergies, gout, psychiatric disorder, or vertigo.

Table S2. Hierarchy applied when multiple reasons for unplanned rehospitalizations coexisted.

**Unplanned cardiac rehospitalizations**
Ischemic events (myocardial infarction and/or stent thrombosis)
Surgical revascularization
Heart surgery due to valvular disease
Heart failure
Recurrent chest pain with ischemia but no intervention
Tachyarrhythmias
Bradyarrhythmias
Pericarditis
Recurrent chest pain without evidence of ischemia
Miscellany
**Unplanned non-cardiac rehospitalizations**
Cerebro-vascular event
Cancer (diagnosis or treatment - not surgery)
Respiratory pathology – not surgical
Major vascular complication (excluding bleeding)
Infections (including sepsis)
Bleeding
Acute renal failure (including decompensation of chronic kidney disease)
Abdominal pathology - not surgical
Non-cardiac surgery: cancer
Non-cardiac surgery: abdominal
Non-cardiac surgery: orthopedic
Non-cardiac surgery: urogenital
Urogenital pathology – not surgical
Non-cardiac surgery: other than the above
Miscellany

Table S3. Hierarchy applied when multiple reasons for planned rehospitalizations coexisted.

**Planned cardiac rehospitalizations**
Surgical revascularization
Heart surgery due to valvular disease
Percutaneous revascularization
Planned implantation of a cardioverter defibrillator
Tachyarrhythmias
Bradyarrhythmias
Miscellany
**Planned non-cardiac rehospitalizations**
Non-cardiac surgery: vascular (aorta, peripheral arteries)
Cancer (diagnosis or treatment - not surgery)
Respiratory pathology – not surgical
Infections
Non-cardiac surgery: cancer (including biopsies)
Non-cardiac surgery: abdominal
Non-cardiac surgery: orthopedic
Non-cardiac surgery: urogenital
Non-cardiac surgery: other than the above (including biopsies not related to cancer)
Urogenital pathology – not surgical
Miscellany

Table S4. Reasons for planned rehospitalizations after PCI for STEMI within one year

**Planned cardiac rehospitalizations**	Nº of RHs	% of RHs
Percutaneous revascularization (any)	8	27.6
Surgical revascularization	7	24.1
Cardioverter defibrillator implantation	7	24.1
Miscellany	5	17.2
Bradyarrhythmias	1	3.5
Heart surgery due to valvular disease	1	3.5
Total	29	100
**Planned non-cardiac rehospitalizations**	Nº of RHs	% of RHs
Surgery: oncology (including biopsies)	8	22.9
Surgery: vascular	7	20.0
Surgery: orthopedics	6	17.1
Neoplasms	5	14.3
Surgery: miscellany	5	14.3
Surgery: urogenital	3	8.6
Miscellany	1	2.9
Total	35	100

RH = rehospitalization

Table S5. Reasons for unplanned rehospitalizations after primary PCI for STEMI separate for patients randomized to bare-metal stent BMS and to biolimus drug-eluting stent BES (including multiple events).

Unplanned cardiac rehospitalizations	BMS № of RHs (n=573)	BMS % of RHs	BES № of RHs (n=564)	BES % of RHs	p-value
Recurrent chest pain without evidence of ischemia*	18	19.15	17	21.79	0.67
Recurrent chest pain with ischemia and subsequent intervention†,‡	22	23.40	7	8.97	0.01
Ischemic events (myocardial infarction and/or stent thrombosis)	17	18.09	12	15.38	0.64
Heart failure	11	11.70	17	21.79	0.07
Recurrent chest pain with ischemia with no coronary intervention†	9	9.57	14	17.95	0.11
Tachyarrhythmias	6	6.38	5	6.41	0.99
Miscellany	7	7.45	2	2.56	0.15
Bradyarrhythmias	3	3.19	2	2.56	0.81
Pericarditis	1	1.06	2	2.56	0.45
Total	94	100	78	100	

Unplanned non-cardiac rehospitalizations	BMS № of RHs (n=573)	BMS % of RHs	BES № of RHs (n=564)	BES % of RHs	p-value
All bleeding	12	26.09	12	23.08	0.73
Miscellany§	9	19.57	7	13.46	0.41
Infections (including sepsis)	6	13.04	8	15.38	0.74
Neoplasms ǁ	3	6.52	6	11.54	0.39
Abdominal pathology ǁ	3	6.52	5	9.62	0.58
Cerebro-vascular event	2	4.35	6	11.54	0.19
Major vascular complication (excluding bleeding)	3	6.52	1	1.92	0.25
Respiratory pathology ǁ	1	2.17	2	3.85	0.63

Urogenital pathology‖	2	4.35	1	1.92	0.49
Surgery: orthopedics	1	2.17	1	1.92	0.93
Surgery: abdominal	1	2.17	1	1.92	0.93
Surgery: miscellany	2	4.35	0	0.00	0.13
Acute renal failure (including decompensation of CKD)	1	2.17	1	1.92	0.93
Surgery: oncology (including biopsies)	0	0.00	1	1.92	0.34
Total	46	100	52	100	

RH = rehospitalization; CKD = chronic kidney disease; BMS = bare-metal stent; BES: biolimus-eluting stent

p-values are derived using the Chi-Square Test for each reason relative to total hospitalizations.

*Among 35 RH, 8 included repeat coronary angiographies, 6 non-invasive stress tests, and 2 non-invasive myocardial perfusion tests.

†Stable or unstable angina.

‡Percutaneous coronary intervention or coronary bypass graft surgery.

§Accident, pupura, pemphigus, drug toxicity, allergies, gout, psychiatric disorder, or vertigo.

‖Diagnosis or non-surgical treatment.

Figure S1. Patient flowchart

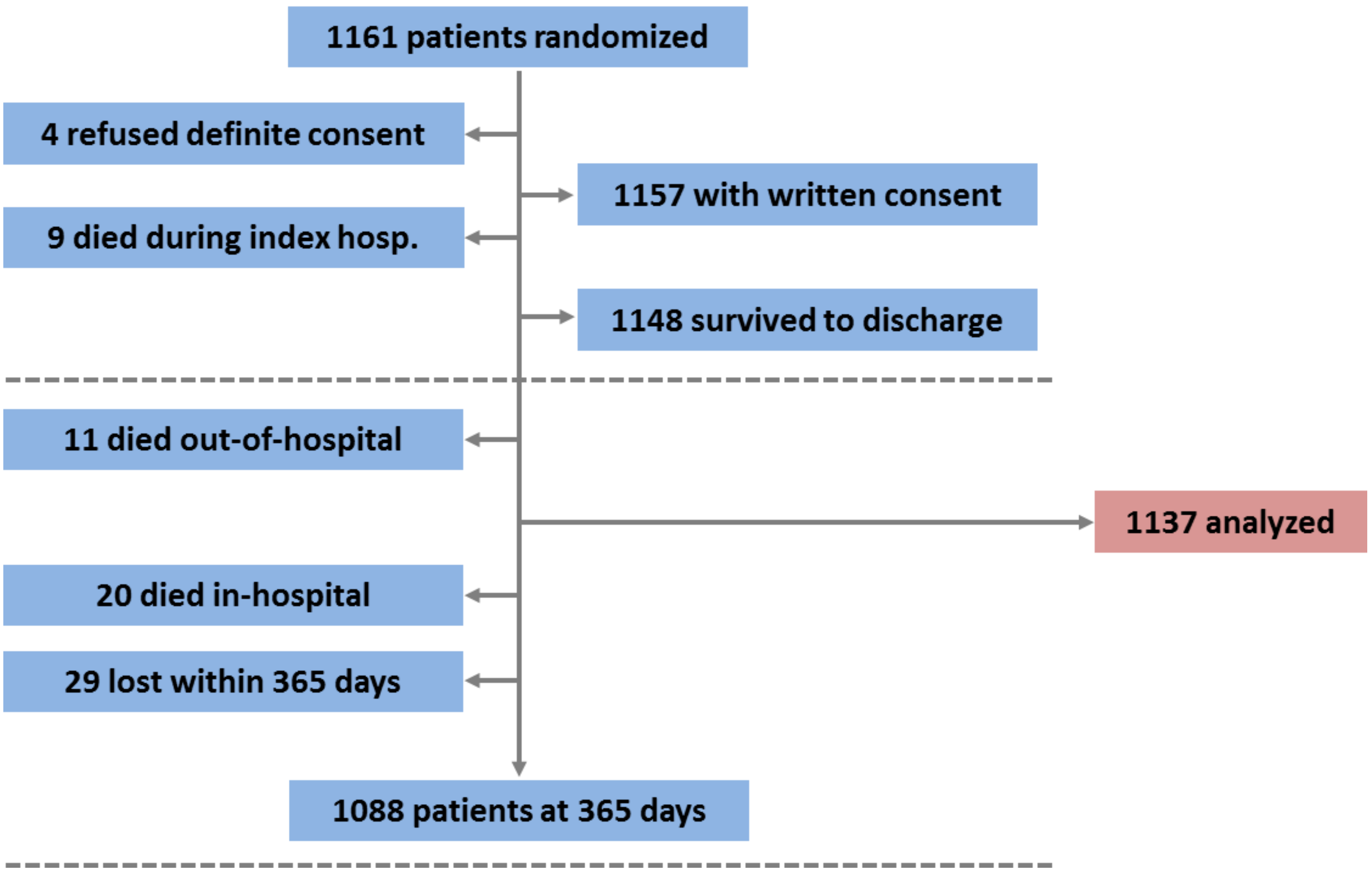

Supplement: Supplementary file 1 — Table S1. List of Reasons for Rehospitalizations Table S2. Hierarchy Applied When Multiple Reasons for Unplanned Rehospitalizations Coexisted Table S3. Hierarchy Applied When Multiple Reasons for Planned Rehospitalizations Coexisted Table S4. Reasons for Planned Rehospitalizations After PCI for STEMI Within 1 Year Table S5. Reasons for Unplanned Rehospitalizations After Primary PCI for STEMI Separate for Patients Randomized to Bare‐Metal Stent BMS and to Biolimus Drug‐Eluting Stent BES (Including Multiple Events) Figure S1. Patient flow chart. [file JAH3-6-e005926-s001.pdf]
